# Biomechanical System Versus Observational Rating Scale for Parkinson’s Disease Tremor Assessment

**DOI:** 10.1038/s41598-019-44142-1

**Published:** 2019-05-31

**Authors:** Ping Yi Chan, Zaidi Mohd Ripin, Sanihah Abdul Halim, Muhammad Imran Kamarudin, Kwang Sheng Ng, Gaik Bee Eow, Kenny Tan, Chun Fai Cheah, Linda Then, Nelson Soong, Jyh Yung Hor, Ahmad Shukri Yahya, Wan Nor Arifin, John Tharakan, Muzaimi Mustapha

**Affiliations:** 1 The Vibration Laboratory, School of Mechanical Engineering, Universiti Sains Malaysia, Engineering Campus, 14300 Nibong Tebal, Penang Malaysia; 2 Department of Medicine, School of Medical Sciences, Universiti Sains Malaysia, Health Campus, 16150 Kubang Kerian, Kelantan Malaysia; 3 Department of Neurosciences, School of Medical Sciences, Universiti Sains Malaysia, Health Campus, 16150 Kubang Kerian, Kelantan Malaysia; 4 Department of Neurology, Penang General Hospital, Residensi Road, 10990 Georgetown, Penang Malaysia; 5 Department of Internal Medicine, Penang General Hospital, Residensi Road, 10990 Georgetown, Penang Malaysia; 6 School of Civil Engineering, Universiti Sains Malaysia, Engineering Campus, 14300 Nibong Tebal, Penang Malaysia; 7 Unit of Biostatistics and Research Methodology, School of Medical Sciences, Universiti Sains Malaysia, Health Campus, 16150 Kubang Kerian, Kelantan Malaysia

**Keywords:** Electrodiagnosis, Neurological manifestations, Biomedical engineering

## Abstract

There is a lack of evidence that either conventional observational rating scale or biomechanical system is a better tremor assessment tool. This work focuses on comparing a biomechanical system and the Movement Disorder Society–sponsored revision of the Unified Parkinson’s Disease Rating Scale in terms of test-retest reliability. The Parkinson’s disease tremors were quantified by biomechanical system in joint angular displacement and predicted rating, as well as assessed by three raters using observational ratings. Qualitative comparisons of the validity and function are made also. The observational rating captures the overall severity of body parts, whereas the biomechanical system provides motion- and joint-specific tremor severity. The tremor readings of the biomechanical system were previously validated against encoders’ readings and doctors’ ratings; the observational ratings were validated with previous ratings on assessing the disease and combined motor symptoms rather than on tremor specifically. Analyses show that the predicted rating is significantly more reliable than the average clinical ratings by three raters. The comparison work removes some of the inconsistent impressions of the tools and serves as guideline for selecting a tool that can improve tremor assessment. Nevertheless, further work is required to consider more variabilities that influence the overall judgement.

## Introduction

The conventional means of tremor assessment is to observationally rate tremor severity using scales. Observational tremor rating scales are considered as gold standards for validating newly developed biomechanical systems^1,2^, which use sensing devices to track and quantify tremors. The correlation or agreement between the rating scores and the biomechanical system readings determines the validity of the system to be evaluated^3–5^.

On the other hand, the biomechanical system is considered as a reference for comparison with observational rating because of its impartiality and precision in the assessment, as well as in providing frequency information^6^. However, the technological advancement does not transcend the barriers of adopting biomechanical system by most clinicians. One of the main reasons for this is that there is lack of evidence in supporting biomechanical system as a more reliable measurement tool. There are studies presenting the test-retest reliability of biomechanical system^7^ and rating scores^8,9^ individually but there is limited work that compares the reliability performance of the two assessment tools. Efforts have been made previously to compare the reliability of tremor quantification of the two assessment tools, but no significant difference was found in the study^10^. The result indicates that both tools can provide reliable readings, and there is no scientific proof that either one of the tools is better.

Additionally, the cost of drug development, which was reported to be more than $1 billion^11^, is mainly attributed to clinical cost^12^. One of the ways to address this concern is to evaluate the conventional and new technologies before selecting a tool that can economically improve a clinical study. Capability and reliability are the key criteria influencing this decision.

In this work, the objective is to provide qualitative and quantitative comparisons between a biomechanical system and the Movement Disorder Society-Sponsored Revision of the Unified Parkinson’s Disease Rating Scale (MDS–UPDRS) in terms of function, validity and test-retest reliability. In the clinic, the tremors of Parkinson’s disease (PD) were measured using the biomechanical system in terms of joint angular displacement and predicted rating; and the video-recorded tremors were assessed using the MDS–UPDRS by three raters. Test-retest study was carried out to provide evidence of the reliability performance of the two tools in measuring PD tremors. The clinical observational ratings and the predicted ratings estimated from the system readings were then assessed and compared for their relative reliability. Qualitative comparisons in terms of the functions and validity presented are important for determining their objectivity and applicability. The comparisons are crucial for the selection of a tremor assessment tool in an application.

The methodology and results center on the test-retest study for providing the evidence for the reliability performance comparison. The qualitative comparisons of the function, means of quantification and the validity are presented in the discussion section.

## Methods

### Tremor measurement system

A hand–arm tremor measurement system was developed to measure multi-degrees-of-freedom (multi-dof) coupled relative motion. The system solves the previous technological problem of not considering coupled motion, which occurs naturally in human motions such as in wrist joint^13^. It also provides information on the location and direction of a tremor, which addresses the concern by Sternberg *et al*. that tremor has been classified in bodily part and there is a lack of assessment of tremor distributed across joint^14^.

The system consists of three units of the triaxial gyro–enhanced Attitude and Heading Reference Systems, model SBG IG–500A (SBG Systems, Rueil–Malmaison, France). In order to consider the coupled motions, the posture at which the related motions are neutral was established. In this posture, the wrist is not flexed, extended, abducted or adducted and the elbow is fully extended without pronation or supination. Each unit of the SBG IG–500A was placed on the hand, upper arm and lower arm, when the upper limb was in this neutral position. The quaternion data from the instruments were acquired and analysed using LabVIEW^TM^ software (National Instruments Corporation, Austin, Texas).

Postprocessing of these data was carried out to compute the joint angle^15^. In this system, the coupling effects of wrist flexion-extension and wrist abduction-adduction, as well as elbow pronation-supination and elbow flexion-extension are considered. When quantifying one of the coupled motions, the axis of rotation at distal segment is corrected using quaternion rotation such that it is parallel to the corresponding axis of the proximal segment. For instance, computing elbow pronation-supination requires the orientation correction of the x-axis of the lower arm so that it is parallel to the x-axis of the upper arm. The joint angle is the angle of rotation for the remaining two axes perpendicular to the corrected rotation axis in the distal segment to align with the corresponding counterparts in proximal segment.

Bandpass filtering (using Butterworth bandpass filter of order four) between 3 and 30 Hz was performed to limit only tremor signals, which are essentially found to be within that range^16^. The resulting parameter of the filtering is termed joint angular displacement, Δθ_joint_, the tremor motion quantification parameter used in the system. The Δθ_joint_ in each of the hand–arm tremor motions, i.e. wrist flexion–extension, wrist abduction–adduction, elbow pronation–supination and elbow flexion–extension was then computed for its root mean square (RMS) throughout the whole measurement before statistical analyses were carried out. The biomechanical system quantification method was previously validated with a high–precision angular encoder system installed on a tremor simulator in addition to being correlated with the doctors’ observational ratings^15^.

### Experimental design

The work in which the test–retest study of measurement system RMS Δθ_joint_ was carried out, involved 61 PD subjects. Forty of these PD patients (PD subgroup) were involved in the observational tremor rating and system predicted rating test–retest study. Comparisons of the test–retest reliability, capability and function of the measurement system and the clinical rating were subsequently made. The overall flow of study and the participation of subjects in each stage are depicted in the supplementary file.

With the approval of the medical research ethics committee, clinical studies were carried out in the Neurology Clinic of the Penang General Hospital, Malaysia. PD patients attending walk–in and appointment clinics were recruited with consent.

The inclusion criteria for all the PD subjects were 40 years old or above, and the diagnosis of the idiopathic PD based on the UK Parkinson’s Disease Society (UKPDS) Brain Bank clinical diagnostic criteria^17^. The exclusion criteria were the intake of substances or drugs that could induce or suppress (except for medication taken for PD) tremors and the presence of tremors causing illness or disease. The clinical characteristics of the subjects are in the supplementary material.

#### Tremor measurement and test–retest procedure

Before measurements, tremor–related illness history and time since the last dose of the tremor–suppressing medication were recorded. The subject was then asked to count the number in decremental order with two per interval and perform resting, outstretching and wing postures for 15 seconds. All the actions were video recorded for the tremor assessment with MDS–UPDRS. Two Logitech high-definition (HD) webcams, model c920 (Logitech, Romanel-sur-Morges, Switzerland) were positioned at right angle and a Sony camcorder, model HDR-SR5E (Sony, Tokyo, Japan) was positioned at an oblique angle to the upper limb measured by tremor measurement system. The videos of the webcams and the measurement data were captured at the same instance in the data acquisition program. The camcorder provided close-up videos of tremor and the operation was synchronised manually with the data acquisition.

The upper limb resting and outstretching postures were performed according to the protocols in MDS–UPDRS upon attainment of permission from the International Parkinson and Movement Disorder Society, and the wing posture was carried out according to the protocol of the Washington Height–Inwood Genetic Study of Essential Tremor Tremor Rating Scale (wTRS).

In order to obtain the test–retest reliability of the measurement system, each of these actions was repeated once immediately after the first trial. The time interval between the two trials was within 1 minute. The short time interval allowed for the measurement to be repeated with minimal introduction of variation of tremor source to fulfil the assumption of the estimation of error that it is due only to the reliability of measurement tool^18^. Since the patients were not recruited upon appointment, some of them had taken tremor–suppressing medication, and the duration between measurement and the intake of the last dose of the medication was variable. All the tremor measurement work was carried out by trained research assistants, and data were processed to obtain the RMS Δθ_joint_ with the post–processing algorithm in LabVIEW^TM^.

#### Rating test–retest procedure

In a previous clinical study, the tremors of PD patients were measured using the same instruments and the same camera settings were applied to record the tremor conditions^15^. Six doctors from the Neurology Units of Penang Hospital and Hospital Universiti Sains Malaysia evaluated the video-recorded tremor using MDS–UPDRS and their ratings were averaged. The biomechanical system readings in RMS Δθ_joint_ were validated with the average ratings of six doctors. The results show that most of the measurement readings can be used to explain the variability of the doctors’ rating in assessing tremor during rest and outstretching postures^15^.

In order to remove the biasness in the rating retest in this study, three research assistants were recruited. They underwent formal and standardised training of MDS–UPDRS and assessed the tremors in resting and outstretching actions on the same subjects. Their ratings were correlated with the previous average ratings of the six doctors from neurology unit for concurrent validation of the clinical rating (refer to supplementary material for more details). The three raters then rated the tremors of two consecutive trials for 40 PD patients in the PD subgroup of this study. The rating was made based on the three viewing angles the video provided and the dimensions of the SBG IG–500A measurement unit were provided as reference to judge the linear displacement observed. The assessments on tremor of the same subject with observational rating were not performed on the same day and the interval to assess the tremors of the two trials was between 1–4 days. The scores of the three raters were averaged for each case. The reliability of average clinical rating was compared with that of the predicted rating measured by the system measured on the same subjects. The retest video sequence was randomised according to random numbers generated by Excel. The raters of the tremor videos were all blinded to the diagnosed disease stage and tremor severity. The rating by each rater was done independently without any communication.

A subset of subjects were involved in the rating test-retest study because the tremor measurement using RMS Δθ_joint_ were part of other larger study, and a sample size of 37 subjects was calculated to be sufficient for carrying out the reliability test (refer to supplementary material for the estimation). Besides, the scope of rating retest is to study tremors in resting and outstretching postures to limit the duration of the study and to reduce the burden of the raters. Within this scope, a total of 156 sets of videos (each with three videos, capturing tremor from different angles), accounted for 156 sets of tremor cases were viewed by each rater. The rating of tremor in wing posture will be studied in future, while the rating of postural tremor in the outreaching posture has been studied.

### Statistical analysis

All the measurement readings, i.e. the RMS of Δθ_joint_, and the ratings were tested for fulfilment of the normality distribution assumption using the Kolmogorov–Smirnov test. Since most of the data were determined to be non–normally distributed, non–parametric statistical tools were selected for the test–retest reliability analyses. The analyses were carried out using IBM SPSS Statistics for Windows version 23.0 (IBM Corp., Armonk, NY, USA) and MATLAB version 2010b (The MathWorks, Inc. Natick, MA, USA). The correlation coefficient and *p* value are presented in 2 decimal places, and the rest of the statistical data are expressed with sufficient decimal places to give standard error of measurement (SEM) one significant digit. When the correlation coefficient data are close to the limits of 1, more decimal place is presented^19^. The formulae for obtaining some of the reliability parameters are supplemented.

#### Test–retest reliability

Prior to the reliability analyses, the predicted ratings in PD subgroup were obtained with the multiple linear regression models relating the system readings of four motions and six doctors’ ratings^15^. For evaluating the test–retest reliabilities of the tools, relative reliability and absolute reliability were analysed. The relative reliability was reported using intraclass correlation coefficient (ICC), and the absolute reliability was expressed in terms of SEM and the minimum detectable change at the 95% CI, confidence interval (MDC_95_).

The parameter ICC is a measure of the degree of correlation and agreement between any two measurements done on a same subject^20,21^. For repeated trials of same subjects, the ICC can be expressed as:

1 $${\rm{ICC}}=\frac{{\rm{PMS}}\,\mbox{--}\,{\rm{EMS}}}{{\rm{PMS}}+(k\,\mbox{--}\,1)\text{EMS}}$$

where PMS is the between-subject mean square, EMS is the within-subject mean square and k is the number of replicated readings taken from any individual subject^22^. From Eq. (1), it is known that the ICC is influenced by the between-subject heterogeneity; thus, presenting the between-subject and within-subject standard deviation in addition to ICC enables judgement of the reliability of different measures of heterogeneity to be made^20^. The presented ICC is the single–measures result of the absolute agreement of the two–way mixed effect model.

The interpretation of ICC values^23^ is as follows:

- <0.40: poor
- 0.40–0.59: fair
- 0.60–0.74: good
- 0.75–1.00: excellent

In order to further understand the values of ICC, the general formula of ICC relating the measurement error and true change^24^ as follows is interpreted:

2 $${\rm{ICC}}=\frac{{\sigma }_{S}^{2}}{{\sigma }_{S}^{2}+{\sigma }_{e}^{2}}\,$$

Based on this formula, ICC is the ratio of true variance, ${\sigma }_{S}^{2}$ to the total variance consisting of both ${\sigma }_{S}^{2}$ and ${\sigma }_{e}^{2}$, which is the variance due to measurement error. A tool with ICC of 1.00 has negligible measurement error as compared to the true variation measured throughout the tested range, whereas a tool with ICC of 0 indicates that the measured variation is almost contributed by the measurement error only and that the actual variation cannot be reproduced.

The MDC_95_ is defined as the smallest change that can be detected by a tool beyond the random measurement error^25^. Within subject standard deviation or SEM is the standard deviation of errors of measurement^26^, which determines the degree of agreement between repeated measurement readings on the same subject. The relation between MDC_95_ and SEM is described as follows:

3 $${{\rm{MDC}}}_{95}={\rm{SEM}}\times 1.96\times \sqrt{2}.$$

The EMS and PMS can be obtained from the one-way repeated-measures ANOVA table in SPSS before deriving the SEM and between-subject SD values using the following equations.

4 $${\rm{SEM}}=\sqrt{{\rm{EMS}}}$$

5 $${\rm{Between}}\,{\rm{subject}}\,{\rm{SD}}=\sqrt{{\rm{PMS}}}$$

Since the distributional assumption cannot be met in some data, bootstrap method was used as the alternative to the analytical method to estimate the 95% CI of reliability values^27,28^. Ten thousand sets of original data were generated with bootstrap before computing the reliability values. The percentile method was then implemented by assigning α and 1-α quantiles of the bootstrap distribution of reliability as the lower and upper confidence limits^27^. An α value of 0.025 was used to find the 95% CI.

For comparing the significant differences in reliability parameters of clinical and predicted ratings, 10,000 ICCs of each rating generated from the previous bootstrap were used^29,30^. A Wilcoxon signed–rank test was then performed to test for significant differences between the predicted and clinical ratings in assessing tremors. The critical value for a 95% CI or 5% level of significance (*p* = 0.05) in the two–tailed test was 1.96. The bootstrapped reliability values of the two tools were made sure to be paired, e.g. each of the sets of ICCs of both ratings was calculated from the same tremor case. In addition to the *p* value of the Wilcoxon signed-rank test, the information as follows is presented:

I. The bootstrap CIs of reliability parameters of each type of rating.
II. The bootstrap CIs of the reliability difference between both ratings.
III. The effect size in eta-square, η^2^ of the Wilcoxon signed-rank test.

The reason for the need of this information is that there are different opinions on the criteria of determining a difference between two estimates. Using an overlap of 95% CIs of two groups as the criteria for no significant difference is incorrect. Two groups are significantly different (*p* < 0.05) if there is no overlapping of the corresponding CIs but the overlapping of CIs does not indicate that there is no significant difference (*p* > 0.05) between the groups^31,32^. However, some medical investigators are interested to determine the size of a difference between groups, instead of only statistical difference and CI with the range of the difference is preferred^33^. The η^2^ of the Wilcoxon signed-rank test also provides indication of the size of the difference. Presenting the aforementioned information satisfies the conditions of different views.

Plots of trial one versus two and Bland–Altman plots for clinical and predicted ratings in PD subgroup are also presented to demonstrate the distribution of the data. Lines indicating the mean difference and limits of agreement (LoA) are marked in the Bland–Altman plots.

#### Impact of reliability on clinical study sample size

The reliability of a measurement tool influences the sample size required in a common clinical study that compares the mean of two groups. Using the model relating reliability based on the ICC and sample size requirement in the study by Perkins *et al*.^34^, the percent reduction in sample size was estimated.

### Ethics approval and consent to participate

All participants read and signed informed consent forms in accordance with ethical guidelines. The Medical Research Ethics Committee (MREC), Secretariat of National Institutes of Health, Malaysia approved this research (trial registration: NMRR-14-1694-21740 (IIR), approved on 14^th^ July 2015).

## Results

### Measurement system reliability

#### Relative and absolute reliabilities

The retest performance in terms of ICC, MDC_95_ and SEM using the RMS Δθ_joint_ to measure tremors in all PD patients is shown in Table 1. The ICC results (Table 1) show that the system has fair to excellent relative reliability (based on ICC value interpretation by Cicchetti, 1994^23^) in measuring PD tremors during resting, outstretching and wing actions. The highest MDC_95_ and SEM in measuring PD tremors are 0.65° and 0.23° (95% CI = 0.05°, 0.37°) respectively.

**Table 1:** Test-retest statistical analyses of measurement system using RMS ${{\rm{\Delta }}{\rm{\theta }}}_{{\rm{joint}}}$ to measure on PD patients.

Action	Motion	ICC (95% CI)	SEM (95% CI)	MDC_95_
Resting (n = 61)	WFE	**0.78** (**0.60, 0.94**)	**0.23** (**0.04, 0.39)**	**0.64**
	WAA	0.92 (0.51, 0.96)	0.14 (0.04, 0.20)	0.38
	EPS	0.92 (0.73, 0.97)	0.18 (0.03, 0.29)	0.50
	EFE	**0.997** (**0.720, 0.999)**	**0.02** (**0.01, 0.03)**	**0.06**
Outstretching (n = 57)	WFE	0.80 (0.46, 0.93)	0.15 (0.05, 0.23)	0.41
	WAA	**0.92** (**0.61, 0.99)**	**0.07** (**0.02, 0.11)**	**0.19**
	EPS	**0.57** (**0.43, 0.91)**	**0.23** (**0.05, 0.37)**	**0.65**
	EFE	0.79 (0.57, 0.98)	0.08 (0.02, 0.12)	0.22
Wing (n = 49)	WFE	**0.951** (**0.886, 0.997)**	0.10 (0.02, 0.17)	0.29
	WAA	0.91 (0.89, 0.97)	**0.07** (**0.02, 0.11)**	**0.20**
	EPS	**0.81** (**0.57, 0.98)**	**0.21** (**0.05, 0.34)**	**0.58**
	EFE	0.91 (0.57, 0.98)	0.09 (0.03, 0.13)	0.24

ICC = intraclass correlation coefficient; SEM = standard error of measurement; MDC_95_ = minimum detectable change; n = number of sample; WFE = wrist flexion-extension; WAA = wrist abduction-adduction; EPS = elbow pronation-supination; EFE = elbow flexion-extension. All the values of the SEM and MDC_95_ are in °. The ICC is the single measures results of the absolute agreement of two-way mixed effect model. The values highlighted in bold are the highest and lowest readings in each parameter.

### Clinical versus predicted ratings (PD subgroup)

**Figure 1:**
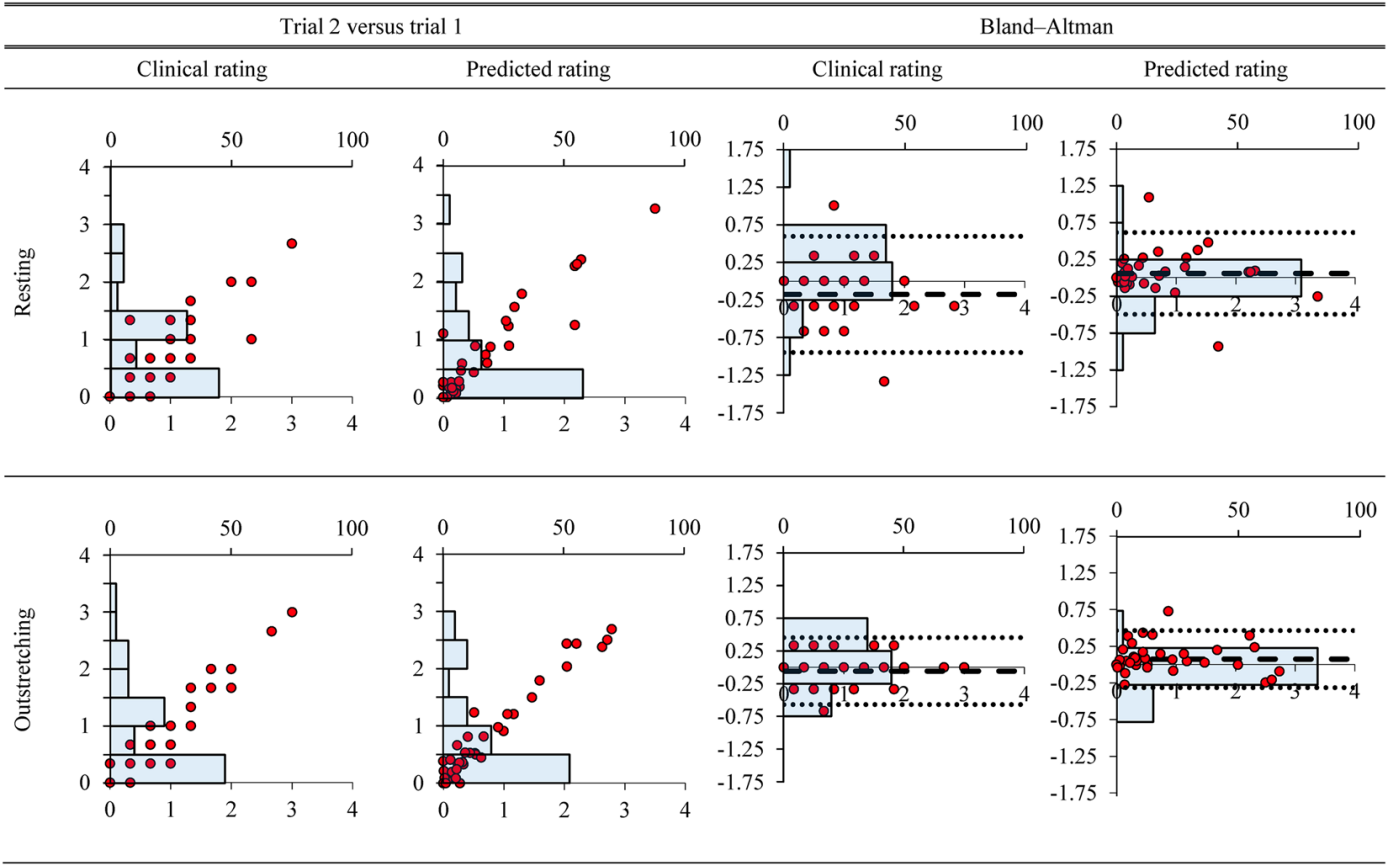
Plots of trial 2 versus trial 1 and Bland–Altman for mean clinical and predicted rating. The y–axis of Bland–Altman plot is the difference between trial 2 minus trial 1 versus the mean difference of the pair trials. The dotted and dashed lines are the limits of agreement and the mean difference. Histograms are overlaid with trial 2 versus 1 plots to show the frequency distribution. The top x–axes are the amount of data in percentage, and the lower x–axes are the ratings. The y–axis of the trial 2 versus trial 1 and Bland–Altman plots are the ratings and rating differences, respectively.

The scatter plots of trial 2 versus trial 1 and Bland–Altman plots for mean clinical and predicted ratings during resting and outstretching postures can be seen in Fig. 1. Since there are many data points are of the same readings, the frequency distributions are presented in histogram and overlaid with the paired trial plots. The plots of trial 2 versus trial 1 and the associated histograms show that the data points representing predicted ratings for both postures are less scattered than those representing the clinical ratings. The Bland–Altman plots of the clinical and predicted ratings in Fig. 1 show uniform variance across the whole range of ratings. The histograms of the clinical rating show more dispersed distributions than those for the predicted ratings. This agrees with the larger LoA obtained by using the clinical rating for assessing both resting and outstretching tremors.

The reliability performances of the clinical and predicted ratings for resting and outstretching actions in terms of SEM, MDC_95_ and ICC are presented in Table 2. The magnitude of the MDC_95_ (and hence SEM) of the predicted ratings in assessing tremors during resting (MDC_95_ = 0.6; SEM = 0.2, 95% CI = 0.1, 0.3) and outstretching postures (MDC_95_ = 0.4; SEM = 0.1, 95% CI = 0.1, 0.2) are lower than the values of all clinical ratings (resting: MDC_95_ = 0.8; SEM = 0.3, 95% = 0.2, 0.4; outstretching: MDC_95_ = 0.5; SEM = 0.2, 95% = 0.1, 0.2).

**Table 2:** Clinical rating and predicted rating test-retest statistical analyses for resting and outstretching postures.

Action	Clinical rating			Predicted rating		
	ICC (95% CI)	SEM (95% CI)	MDC_95_	ICC (95% CI)	SEM (95% CI)	MDC_95_
Resting (n = 38)	0.85 (0.68, 0.93)	0.3 (0.2, 0.4)	0.8	0.94 (0.83, 0.98)	0.2 (0.1, 0.3)	0.6
Outstretching (n = 40)	0.92 (0.85, 0.96)	0.2 (0.1, 0.2)	0.5	0.97 (0.93, 0.99)	0.1 (0.1, 0.2)	0.4

ICC = intraclass correlation coefficient; SEM = standard error of measurement; MDC = minimum detectable change; n = number of sample; WFE = wrist flexion-extension; WAA = wrist abduction-adduction; EPS = elbow pronation-supination; EFE = elbow flexion-extension. All the values of the SEM and MDC_95_ are in °. The ICC is the single measures results of the absolute agreement of two-way mixed effect model. The 95% CI of ICC were obtained from the 10,000 sets of ICC generated from bootstrap method.

Similarly, the ICCs of predicted rating in resting (ICC = 0.94, 95% CI = 0.83, 0.98) and outstretching (ICC = 0.97, 95% CI = 0.93, 0.99) are higher than those of clinical rating (resting: ICC = 0.85, 95% CI = 0.68, 0.93; outstretching: ICC = 0.92, 95% CI = 0.85, 0.96). In other words, ICC values of 0.85 and 0.94 indicate that 85% and 94% of the total variation is due to actual variation when using clinical and predicted ratings to measure resting tremor. In measuring postural tremor using the clinical and predicted rating, ICC values of 0.92 and 0.97 can be interpreted such that 92% and 97% of the total variation is due to actual variation.

The 95% CI of SEM and ICC for both ratings overlap in every posture. Further analysis results of comparing the significant difference of 10,000 sets of ICC are tabulated in Table 3. There were 10,000 sets of ICC generated using bootstrapping for the reliability comparative statistical analysis. The results show that the ICC values of the clinical ratings in resting and outstretching are significantly lower than the predicted ratings in resting (Z = −86.6; *p* < 0.0001, Wilcoxon signed–rank test) and outstretching postures (Z = −86.4; *p* < 0.0001, Wilcoxon signed–rank test). The effect of the difference in ICC is large (η^2^ = 0.75) based on the interpretation guidelines by Cohen (1988) (small effect η^2^ = 0.01, medium effect η^2^ = 0.06 and large effect η^2^ = 0.14)^35^.

**Table 3:** Significant difference between the relative reliability values of predicted and clinical ratings.

	ΔICC (95% CI)	ICC	
		Z (*p* value)	η^2^
Resting	0.09 (0.03, 0.18)	−86.6 (<0.0001)	0.75
Outstretching	0.05 (0.004, 0.101)	−86.4 (<0.0001)	0.75

ICC = intraclass correlation coefficient; ΔICC = ICC predicted rating – ICC clinical rating. The *p* value contains the information of the significance level of the difference between the 10,000 pairs of reliability values of predicted and clinical ratings generated from Wilcoxon signed rank test (two-tailed). The Eta-squared, η2 indicates the effect size.

Apart from this, the ΔICC value, denoting the ICC of predicted rating minus the ICC of clinical rating in each tested posture is presented in Table 3. The 95% CI ΔICCs of both resting and outstretching postures are from 0.03 to 0.18, and from 0.004 to 0.101 respectively. These intervals are all above zero and positive. The results of not including zero difference within 95% CI of ΔICC agree with the *p* values of Wilcoxon signed-rank tests to indicate significant differences between the ICCs of the two tools in assessing both resting and outstretching postures. The positive ΔICC values in all cases indicate that the ICCs of the predicted rating are higher than those of the clinical ratings. The 95% CI of ΔICC further reveals that the population value of the difference in relative reliability may be from as small as 0.004 (for resting) and 0.03 (for outstretching) to 0.101 (for outstretching) and 0.18 (for resting). Nonetheless, the population ΔICC is more likely to be in between of the CI, i.e. 0.09 and 0.05 for resting and outstretching respectively, where 0.15 to 0.25 change in ICC marks a change in the level of reliability based on the interpretation by Cicchetti, 1994^23^.

### Impact of reliability on clinical study sample size

**Figure 2:**
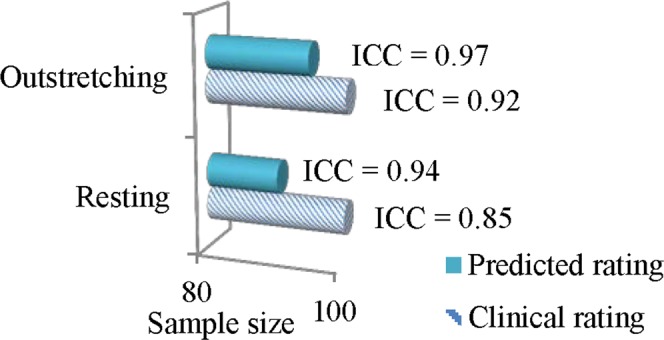
Impact of reliability on the sample size required for clinical studies. ICC = intraclass correlation coefficient; n = number of subjects.

The impact of relative reliability on the clinical study sample size is illustrated in Fig. 2. In a resting posture, the higher ICC of the predicted rating is estimated to reduce the sample size by 9.6% as compared to using the clinical rating. In an outstretching posture, the percent reduction using the predicted rating instead of the clinical rating is 5.2%. In Fig. 2, 100 subjects for the clinical rating are used as a reference to illustrate the impact of sample size reduction. A clinical study using the predicted rating requires 90 and 95 subjects in resting and outstretching respectively.

## Discussion

**Figure 3:**
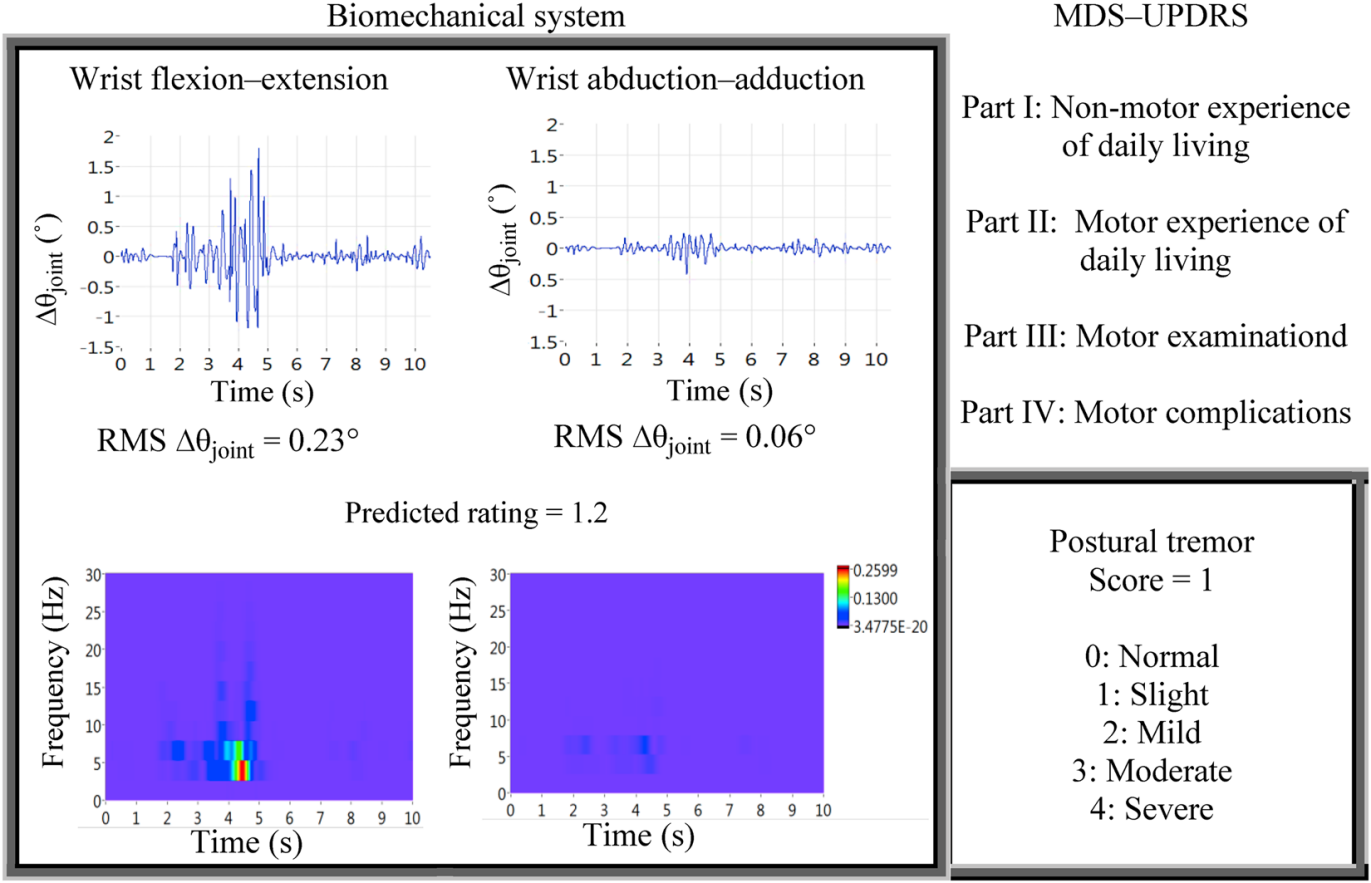
Outputs of biomechanical system versus clinical observational rating. The data were taken from the tremor measurement of a PD patient with outstretching hand.

In this section, the comparisons between the MDS-UPDRS and the biomechanical system developed are discussed based on the following aspects: (i) the function and means of quantification, (ii) the means of validation and the validity performance and (iii) the test-retest reliability performance. The function and the means of quantification determine the objectivity and applicability of the tools. Figure 3 depicts the functions of the MDS-UPDRS and biomechanical system. MDS-UPDRS is a multidimensional scale that consists of four parts to assess non-motor and motor symptoms of PD^36^. Tremor assessment within the motor examination (Part III) has clear and specific instruction to carry out the examination that enables the standardization of rating process. The way the assessment has been pre-structured according to the conditions in which a tremor occurs is useful in understanding a tremor because such behavioral characteristics are often used as the criteria to classify tremor etiology^37^ though not exclusively and distinctively.

The tremor data in Fig. 3 were taken when a PD subject was outstretching upper limb. The 5-point rating scale of MDS-UPDRS gives the overall tremor severity of body parts, whereas the biomechanical system provides motion– and joint–specific tremor severity. The overall apparent hand–arm tremor amplitude observed is a manifestation of multi–dof coupled motion. Under certain conditions, motions of connected segments interfere with one another such that the amplitude at a segment is amplified or reduced, causing the perceived tremor not to reflect the amplitude of involuntary muscle contractions. One such example is when the upper limb is in outstretching posture based on the MDS-UPDRS protocol, the tremor amplitude in wrist flexion-extension interferes constructively or destructively with the tremor in elbow flexion-extension, when the components of rotation axis are orientated parallel to each other. Nevertheless, the overall resultant interactions of segmental motion are still captured within observational rating, and the selection of the tool depends on the intended outcomes of a study and the underlying assumption of interpreting the parameters of the assessment tool.

It is crucial to understand the function of an assessment tool because it determines the characteristics of a tremor that can be studied. Based on Fig. 3, the five ratings of MDS-UPDRS are designed for easy interpretation and communication as compared to real physical quantification performed by the biomechanical system, i.e. in degrees. The MDC_95_ is required as a reference to judge the relative severity based on two measurement readings. Apart from this, the outputs of biomechanical system include times series of Δθ_joint_ (top left of Fig. 3) and the frequency spectrum (bottom left of Fig. 3) in each tremor motion. Based on the temporal information, the change in both amplitude and frequency of the tremor can be analysed and documented, in contrast to observational and predicted ratings, which quantify maximum amplitude without providing information on the duration of the amplitude. In short, MDS-UPDRS tremor rating provides severity assessment of standard behavioural characteristics, applicable for symptom progression assessment and to be used as the endpoint of an intervention and/ or disease. The biomechanical system is suitable for similar application and can further provide more detailed and versatile analysis for studying tremor from different perspective.

The MDS-UPDRS was established to improve the UPDRS. Thus, the validation was done by correlating the total score (Pearson correlation coefficient, r = 0.96) and individual parts (For Part III, r = 0.96) of the two rating scales^36^. The total UPDRS score was previously validated against the Hoehn and Yahr staging. The criterion validity was found to be 0.71 based on the Spearman rank correlation coefficient. Tremor, specifically was found not to be correlated with any other factors in the UPDRS. This indicates that tremor is independent of the severity of PD assessed based on other factors^38^.

On the other hand, the biomechanical system developed was validated against rotary encoder systems mounted on a tremor simulator^15^. The standard error of estimate and coefficient of determination obtained from linear regression relating the RMS of angular displacement, Δθ of the two systems are 0.03° (95% CI = 0.03°, 0.04°) and 1.000 (p < 0.001) respectively. Further regression analysis shows that the system readings are able to explain more than 64% variability of six doctors’ rating in drinking actions and above 80% variability of doctors’ rating in resting, outstretching and wing actions (MDS-UPDRS was used to assess tremors of resting and outstretching actions; wTRS was used to rate tremors of wing posture and drinking actions)^15^. In essence, the biomechanical system has been tested more intensively in measuring tremor, whereas the validation of MDS-UPDRS focused on assessing the disease as a whole and limited information was found on validating the tremor score.

Reliability is another important aspect to consider when selecting a tremor measurement tool. A more reliable tool is marked by less random variation during repeated tests, as indicated by less dispersed data points in the scatter and Bland–Altman plots, usually with the support of lower SEM values. The lower measurement error (in SEM) enables a tool to detect smaller change in a characteristic of a subject, as indicated by lower MDC_95_ values. The ICC value of a reliability test delineates the relation of the measurement error with the tested range of measurement readings. For the case of testing the reliability of two tools in measuring the same samples, a tool with higher ICC is desired because it suggests that the tool has measurement error that is much lower than the true variation of interest.

Through comparing the reliability of clinical and predicted ratings, the statistical findings are summarised as follows: The scatter plots of trial 2 versus trial 1 and Bland–Altman plots are less scattered for the predicted ratings than for clinical ratings, as supported by the lower values of SEM and MDC_95_. Moreover, the ICC is greater when using predicted rating in assessing tremor. The analysis on the ΔICC and ICC shows that the ability of predicted rating in maintaining the order of severity during repeated measurements is significantly greater than that of clinical rating. The predicted rating has ICC values that are 0.09 and 0.05 higher than those of clinical rating in measuring resting and outstretching postures. In other words, by using the predicted rating, the true variation over total variation can be detected has increased 9% and 5% respectively under the tested conditions. Based on the Wilcoxon signed-rank test, the effect on the change in ICC due to the choice of tool in each ease is large. The effect size in η^2^ is useful to be served as a reference for comparison of reliability performance with other technology or future study^39^.

The clinical implication of this study is that all the reliability parameters agree that the reading can be more reproducible with predicted rating as compared to clinical ratings when measuring tremor repeatedly under the tested conditions. This suggests that smaller change of tremor, that may not be noticed during clinical observational rating may be traced with the predicted rating of biomechanical system but further study is still required to confirm whether it is able to push the envelope of understanding the characteristics of Parkinson’s disease tremor. Using the predicted ratings of the same measurement system at different medical centres is expected to make the tracking of tremor progression, one of the cardinal symptoms of PD^40^, more standardised. Since the system has previously been validated with the doctors’ ratings^15^ it can be used for providing a second opinion in tremor severity assessment, particularly under conditions when tremors are small or difficult to rate. The use of the system outside the clinic can in fact enable the monitoring of the response of tremor medication more objectively as compared to subjective reports by the patient. Moreover, the comparison results preliminarily suggest that the higher ICC of the measurement system under the reported test-retest measurement condition contributes to the reduction in the sample size for carrying out each tremor assessment–related research.

Nevertheless, direct comparisons of relative physical tremor severity using ratings are not easily made. This is because the magnitude difference in actual tremor amplitude from score 1 to 2 is different to that from score 2 to 3, since the ratings are logarithmically related to the actual amplitude^41^. Fitting four to five rating scores to all tremors is also limited in finding subtle change, which is particularly important in finding the individualised optimum amount of medication sufficient to suppress the tremor, yet can prevent the occurrence of drug–induced dyskinesia^42^. Quantifying tremors in continuous number such as RMS Δθ_joint_ eliminates this problem while maintaining the actual physical condition of the severity. Based on the analysis, the reliability values in measuring four hand-arm tremor motions are slightly different and the highest MDC_95_ is 0.64°. In other words, the level of subtlety the system can measure is comparable with 1/10 of a tick of a second hand in a clock, which is 0.6°. In addition, the chronological data of the amplitude and frequency provided by the system developed can be used to study the transient nature of tremors as compared to a rating score.

The previously reported retest showed fair to excellent reliability in using the UPDRS tremor subscale (ICC = 0.63–0.82)^8^ and a very strong correlation between two trials in using the wTRS (regression correlation coefficient, R = 0.98)^9^. As for the retest measurement using biomechanical systems such as TREMBAL, an electromagnetic motion tracking system, the results showed excellent reliability in measuring both translational (ICC > 0.80) and rotational amplitudes (ICC > 0.90)^7^, and the computerised rating score generated from an inertial–measurement–unit–based system showed good to excellent reliability (resting tremor ICC = 0.68, postural tremor ICC = 0.71)^10^. These results are comparable to the predicted rating and RMS Δθ_joint_ ICC found in this study. The reliability results presented in this work enables the performances between conventional and new tools to be directly compared and the results further break the anecdotal impressions of which tool is more reliable.

The clinical values after being averaged are not in integers but they still have the intrinsic discrete nature. Due to the discrete nature, larger variation is expected in the clinical rating as compared to the predicted rating of biomedical system but significant difference between the reliability (in terms of ICC) of the two ratings was not found in the previous study^10^, as contrary to the findings in this study.

The significantly higher relative reliability of the predicted rating as compared to the observational rating suggests that the system developed can supplement the state–of–the–art system known as Kinesia, which was tested with the similar retest procedure to have significantly higher reliability and sensitivity to change than clinical ratings for assessing motor symptoms of PD (bradykinesia, hypokinesia and dysrhythmia) other than tremors^10^. Furthermore, the novel capability of the system in quantifying tremors in multi-dof relative motion will open up new avenues of tremor research.

The study of comparing the conventional and biomechanical system reliability were analysed based on different interpretations of statistics. The Wilcoxon signed-rank test result was supported by the bootstrap CIs of the reliability difference between both ratings to indicate a significant difference and the size of the difference. The effect size of the difference is also indicated by the η^2^ value. These multiple analyses results are presented to satisfy readers of different perspectives to interpret the relative performance of the two tools.

Based on the intended objective of the study to assess the overall performance (rather than performance based on the sub-severity of tremor) of the two tools in PD tremors in general, the patients were not recruited based on severity criterion. Most of the patients recruited had relatively low tremor by chance. This implies that the analysis findings are limited to such distribution of tremor severity. Clearer conclusion on the relative performance for each severity can be drawn if more patients of greater severity are recruited in future. Nevertheless, low tremor which was reported by doctors to be more difficult to be rated is in fact more important in the relative performance comparison study because any potential superiority in the performance of the non-observational tool can ease the doctors’ problem in tremor assessment.

Further studies are required to address the concerns of the clinicians to adopt new technologies. Improvement can be made in the reliability comparison by adding in the inter-rater variability and variability when the system is operated by different personnel and in random sequence of mounting the sensing unit. Further studies can be done for the cost-benefit analysis by considering the expenses and time due to operation, training, function and performance of each tool. Our work has contributed to part of the analysis and other researchers are welcomed to provide other information to help clinicians in deciding whether there is a need to change the conventional means of assessing tremor.

## Conclusion

In short, the MDS–UPDRS provides easily interpretable tremor severity rating, which is suitable for the overall body part assessment; whereas the biomechanical system presented herein, quantifies tremor in joint angular displacement that enables the characterization in specific tremor motion. The predicted rating derived from the displacement has ICC 0.09 and 0.05 higher than that of the clinical rating during retest in resting and outstretching postures. The joint angular displacement which was found to have fair to excellent reliability in the clinical context is in fact more favourable for analysing physical severity compared to rating in general. The advantage of the biomechanical system over observational rating in providing information on the joint location, hand–arm motion, chronological data and predicted rating is expected to benefit clinicians in PD tremor assessment and study. In terms of validity, the biomechanical system was tested more intensively in the laboratory and in the clinical study as compared to the MDS-UPDRS. The presented comparison work provides evidence that breaks part of the inconsistent impressions of the observational ratings and biomechanical system. The presented work serves as a guideline for the tool selection for the purpose of improving the tremor assessment, while supporting that the overall well-established protocol of PD assessment of MDS-UPDRS shall be maintained. Nevertheless, further study is required to support whether the system is better and worth to be adopted in clinical practice.

## Supplementary information

Additional materials

## Data Availability

The dataset used and/or analysed during the current study available from the corresponding author on reasonable request.
