# Supplementary material for: Biomechanical System Versus Observational Rating Scale for Parkinson’s Disease Tremor Assessment
Source: Sci Rep. 2019 May 31;9:8117. doi: 10.1038/s41598-019-44142-1 (PMC6544817; doi:10.1038/s41598-019-44142-1)
Supplement: Supplementary file 1 — Additional materials [file 41598_2019_44142_MOESM1_ESM.pdf]

Biomechanical System Versus Observational Rating Scale for Parkinson's Disease Tremor Assessment

Ping Yi Chan[1*], Zaidi Mohd Ripin[1], Sanihah Abdul Halim[2], Muhammad Imran Kamarudin[2], Kwang Sheng Ng[3], Gaik Bee Eow[4], Kenny Tan[4], Chun Fai Cheah[4], Linda Then[4], Nelson Soong[5], Jyh Yung Hor[4], Ahmad Shukri Yahya[6], Wan Nor Arifin[7], John Tharakan[3], Muzaimi Mustapha[3]

*Corresponding: cpingyi@hotmail.com

[1]The Vibration Laboratory, School of Mechanical Engineering, Universiti Sains Malaysia, Engineering Campus 14300 Nibong Tebal, Penang, Malaysia.

[2] Department of Medicine, School of Medical Sciences, Universiti Sains Malaysia, Health Campus, 16150 Kubang Kerian, Kelantan, Malaysia.

[3] Department of Neurosciences, School of Medical Sciences, Universiti Sains Malaysia, Health Campus, 16150 Kubang Kerian, Kelantan, Malaysia.

[4] Department of Neurology, Penang General Hospital, Residensi Road, 10990 Georgetown Penang, Malaysia.

[5] Department of Internal Medicine, Penang General Hospital, Residensi Road, 10990 Georgetown Penang, Malaysia.

[6] School of Civil Engineering, Universiti Sains Malaysia, Engineering Campus 14300 Nibong Tebal, Penang, Malaysia.

[7] Unit of Biostatistics and Research Methodology, School of Medical Sciences, Universiti Sains Malaysia, Health Campus, 16150 Kubang Kerian, Kelantan, Malaysia.

E-mail addresses:
PYC: cpingyi@hotmail.com
ZMR: mezaidi@usm.my
SAH: sanihahabdhalim@yahoo.com
MIK: imran.kama@gmail.com
KSN: kwangsheng32@gmail.com
GBE: eowgb@yahoo.com
KT: tankenny80@yahoo.com
CFC: highhand@hotmail.com
LYY: lindathen83@gmail.com
NS: soongfs@gmail.com
JYH: horjy@yahoo.com
ASY: ceshukri@usm.my
WNA: wnarifin@usm.my
JT: jtbt7@hotmail.com
MM: mmuzaimi@usm.my

Additional materials

A. Clinical study flow and subject participation

The overall flow of study and the participation of subject in each stage are as depicted in Fig. S1. There were 65 PD patients who were eligible based on the inclusion and exclusion criteria recruited for this and other larger studies. Three patients whose measurement data were lost and one patient who was found to have dyskinesia were excluded from any analysis. Since the system has not been tested for excluding dyskinesia from the tremulous data, eliminating the data removes any potential error from being introduced into the study. Of the remaining 61 patients' data included in the RMS $\Delta\theta_{joint}$ test-retest study, 40 patients were randomly selected for the clinical and predicted ratings test-retest study. Each of the raters assessed all the 40 subjects and the averages of the three ratings were compared with the system-generated predicted ratings.

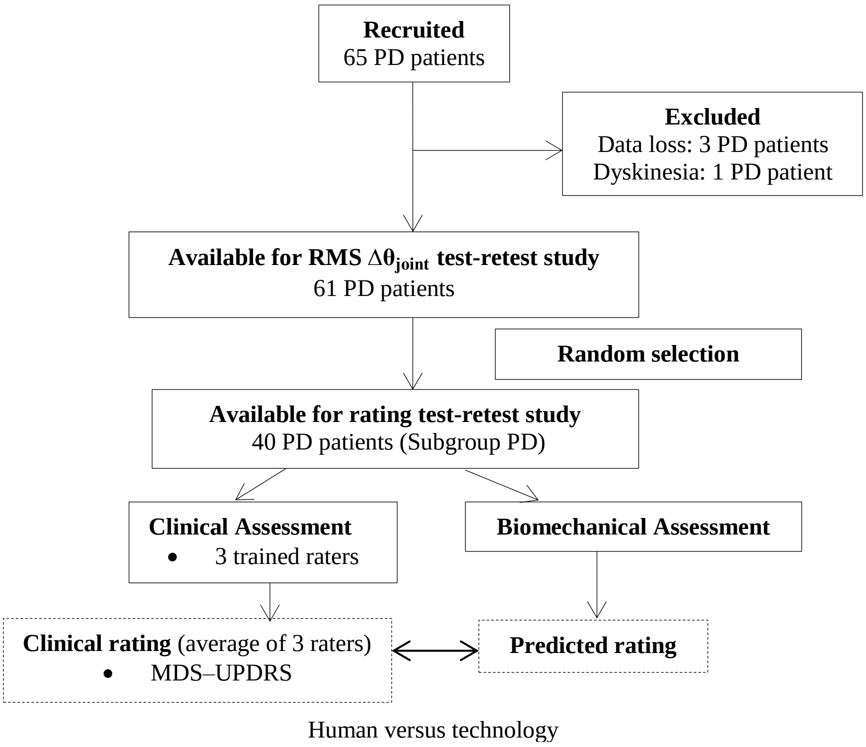

Figure S1. Patient participation and test-retest study flow diagram.

The number of available and missing data for $\Delta\theta_{joint}$ and rating test-retest analyses is as tabulated in table S1. The missing data in some actions of the variables were due to the refusal of the participants and time constraints available for the measurement. The number of each category of the non-participants was not available. All the missing data were not manipulated.

Table S1. Number of available and missing data for each tested variable

Parameter		Rest	Outstretching	Wing
RMS $\Delta\theta_{joint}$	Data available	61	57	49
	Data missing	0	4	12
	Total	61	61	61
Rating	Data available	38	40	-
	Data missing	2	0	-
	Total	40	40	-

B. Clinical characteristics of subjects

The mean ages of the 61 Parkinson's disease subjects involved in the measurement retest study were 70.0 years, respectively (Table S2). The age range of the subjects is 50–86 years old. The percentage value of males was slightly higher than those of females (62.3%). The subjects varied in duration since the last intake of medication, which affected the severity of the tremors measured. An estimated medication wear-off period of 3 hours was used as a reference point to characterize the patients recruited. There was a relatively greater number of subjects who had taken medication 3 hours or more before the assessment (n = 47; 77.0%). Five (6.9%) subjects who could not report that duration were categorised as unknown for that criterion.

Table S2 Clinical characteristics of all the subjects involved in the clinical studies for comparison of tremor characteristics

		Parkinson's disease
		Resting, outstretching, wing (n = 61)
Mean age (SD; range)		70.0 (8.3; 50–86)
Male, number (%)		38 (62.3)
Duration since last medication intake, number (%)	<3 hours	9 (14.8)
	≥3 hours	47 (77.0)
	unknown	5 (8.2)

C. Statistical analyses

C.1 Effect Size

The effect size is defined as "any statistic that quantifies the degree to which sample results diverge from the expectations specified in the null hypothesis"[1]. In this study, it is used to judge the practical significance of the reliability parameter presented. The effect size of Wilcoxon-signed rank test for a measure of significant difference can be represented in eta-square, η^2. The η^2 is computed using the formula as follows[2]:

$$\eta^2 = \frac{z}{\sqrt{N}} \qquad (3)$$

The z value is obtained from the Wilcoxon signed rank test, whereas N is the total sample size.

C.2 Impact of reliability on clinical study sample size

Using the model relating reliability based on the ICC and sample size requirement in the study by Perkins et al. (2000), the percent reduction in sample size can be estimated using the formula below:

$$100\times\frac{ICC_H - ICC_L}{ICC_H} \quad (3)$$

Where ICC_H = ICC of higher reliability tool; ICC_L = ICC of lower reliability tool. In this work, the ICC_H and ICC_L are taken from the median values of the 1000 ICC obtained by bootstrapping of the clinical and predicted ratings.

D. Correlation between the trained raters' and the doctors' clinical ratings

In order to remove the biasness in observational rating, three research assistants were recruited in this study. All of them underwent similar formal and standardized training of MDS-UPDRS rating.

For concurrent validation, the ratings of three individual raters were correlated to the average six doctors' ratings obtained from the previous study.[3] These doctors were in the neurology unit who assessed Parkinson's disease patients on a regular basis. The correlation results are in Supplementary Table S3. The Spearman rank correlation coefficient, ρ of the three raters ranged from 0.63 to 0.67 in an outstretching posture (average rating = 0.69), whereas in a resting posture, the range of ρ was slightly higher, i.e. from 0.75 to 0.87 (average rating = 0.88). The strong to very strong correlation of individual raters' and doctors' ratings support the use of the ratings of the three trained raters for the assessment of test–retest reliability.

Besides, the test-retest reliability of the raters in this work is also comparable with the performance of the raters in other studies in terms of ICC (resting tremor: 0.63 – 0.80[4,5]; postural tremor: 0.68[5]; action tremor: 0.51-0.5[4]) and MDC (resting tremor: 0.85[5]; postural tremor: 0.82[4]). The test-retest reliability of the three raters as compared to raters in other studies is as shown in Table S4.

Table S3 Correlation between the ratings of the trained personnel and the doctors.

	P1	P2	P3	Mean
Resting	0.87	0.75	0.77	0.88
Outstretching	0.67	0.66	0.63	0.69

The values are the Spearman rank correlation coefficient, ρ; P1-3=trained personnel 1-3.

The interpretation of the Spearman rank correlation is as follows[6]:

- 0.80-1.00: very strong
- 0.60-0.79: strong
- 0.40-0.59: moderate
- 0.20-0.39: weak

- 0.00-0.19: very weak

Table S4 The test-retest performance of the raters in this and other studies.

Rater	ICC		MDC_{95}	
	Resting tremor	Action tremor	Resting tremor	Action tremor
Three trained research assistants	0.85	0.92	0.8	0.5
Two movement disorders specialists[5]	0.63	0.68	0.85	0.82
One movement disorder specialist[4]	0.74-0.80	0.51-0.54	-	-

E. Study size estimation

The tremor measurements using RMS $\Delta\theta_{joint}$ were part of other larger study, whereas the sample size, N of the observational clinical ratings test and retest study was estimated using PASS software version 16.0.2 (NCSS, LLC, Kaysville, Utah, USA). For estimating the sample size of obtaining ICC from 0.63 to 0.98 as reported in previous work of studying the retest reliability using UPDRS tremor subscale [7] and wTRS [8] and by expecting that there is at least a fair correlation, i.e. ICC of 0.4 based on the interpretation by Cicchetti, 1994 [9], the following values are assigned:

Power: 0.8

Number of observations per subject):2

$\rho0$: 0.4

$\rho1 > \rho0$: 0.63 – 0.98

where,

Power is the probability of rejecting a false null hypothesis.

$\rho0$ is ICC of assuming the null hypothesis.

$\rho1$ is ICC of assuming the alternative hypothesis.

Fig. S2 shows the results of PASS in estimating the N based on different $\rho1$. Increasing $\rho1$ reduces the N estimated for a study and the maximum value of N is found to be 37 for $\rho1$ of 0.63. A sample size of 40 subjects is used for the test-retest study of ratings.

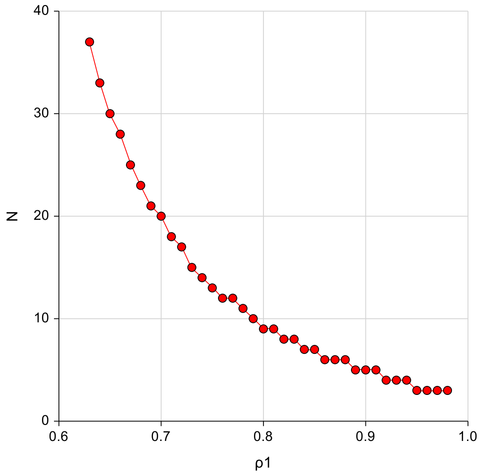

Figure S2. N versus ρ1.

N = sample size; ρ1 = ICC of assuming the alternative hypothesis

References

1 Sun, S., Pan, W. & Wang, L. L. A comprehensive review of effect size reporting and interpreting practices in academic journals in education and psychology. *J Educ Psychol* **102**, 989 (2010).

2 Fritz, C. O., Morris, P. E. & Richler, J. J. Effect size estimates: current use, calculations, and interpretation. *Journal of experimental psychology: General* **141**, 2 (2012).

3 Chan, P. Y. et al. An in–laboratory validity and reliability tested system for quantifying hand–arm tremor in motions. *IEEE Trans. Neural Syst. Rehabil. Eng*. 26, 460–467 (2018).

4 Post, B., Merkus, M. P., de Bie, R. M., de Haan, R. J. & Speelman, J. D. Unified Parkinson's disease rating scale motor examination: are ratings of nurses, residents in neurology, and movement disorders specialists interchangeable? *Movement Disorders: Official Journal of The Movement Disorder Society* **20**, 1577-1584 (2005).

5 Heldman, D. A., Espay, A. J., LeWitt, P. A. & Giuffrida, J. P. Clinician versus machine: reliability and responsiveness of motor endpoints in Parkinson's disease. *Parkinsonism Relat Disord* **20**, 590-595 (2014).

6 Owen, A. Spearman’s correlation. <http://www.statstutor.ac.uk/resources/uploaded/spearmans.pdf>.

7 Siderowf, A. *et al.* Test–retest reliability of the unified Parkinson's disease rating scale in patients with early Parkinson's disease: results from a multicenter clinical trial. *Mov Disord* **17**, 758-763 (2002).

8 Louis, E. D., Ford, B. & Bismuth, B. Reliability between two observers using a protocol for diagnosing essential tremor. *Mov Disord* **13**, 287-293 (1998).

9 Cicchetti, D. V. Guidelines, criteria, and rules of thumb for evaluating normed and standardized assessment instruments in psychology. *Psychol assess* **6**, 284 (1994).
